# How Do Children Behave Regarding Their Birth Order in Dental Setting?

**Published:** 2015-12

**Authors:** Faezeh Ghaderi, Soleiman Fijan, Shahram Hamedani

**Affiliations:** a Dept. of Pediatrics, School of Dentistry, Shiraz University of Medical Sciences, Shiraz, Iran.; b Dept. of Pediatrics, School of Dentistry, Rafsanjan University of Medical Sciences, Rafsanjan, Iran.; c DDS, MSc, Dental Research Development Center, School of Dentistry, Shiraz University of Medical Sciences, Shiraz, Iran.

**Keywords:** Behavior, Birth order, Anxiety

## Abstract

**Statement of the Problem:**

Prediction of child cooperation level in dental setting is an important issue for a dentist to select the proper behavior management method. Many psychological studies have emphasized the effect of birth order on patient behavior and personality; however, only a few researches evaluated the effect of birth order on child’s behavior in dental setting.

**Purpose:**

This study was designed to evaluate the influence of children ordinal position on their behavior in dental setting.

**Materials and Method:**

A total of 158 children with at least one primary mandibular molar needing class I restoration were selected. Children were classified based on the ordinal position; first, middle, or last child as well as single child. A blinded examiner recorded the pain perception of children during injection based on Visual Analogue Scale (VAS) and Sound, Eye and Movement (SEM) scale. To assess the child's anxiety, the questionnaire known as “Dental Subscale of the Children's Fear Survey Schedule” (CFSS-DS) was employed.

**Results:**

The results showed that single children were significantly less cooperative and more anxious than the other children (*p*<0.001). The middle children were significantly more cooperative in comparison with the other child's position (*p*< 0.001).

**Conclusion:**

Single child may behave less cooperatively in dental setting. The order of child birth must also be considered in prediction of child’s behavior for behavioral management.

## Introduction

Many researches have been enrolled to evaluate the effect of different factors on children behavior. It is essential for a pediatric dentist to recognize child’s mood, personality and subsequently the child’s behavior during a dental procedure. Frankl *et al.*[1] (1962) classified children based on their mood and behavior in a dental visit. The behavioural rating scale offered by Frankl is described as definitely negative (when the child is completely uncooperative, crying, very difficult to make progress); negative (when the child is uncooperative, very reluctant to listen/respond to questions, some progress possible); positive (when the child is cooperative, somewhat reluctant/shy), and definitely positive (when the child is completely cooperative and even enjoys the experience).[1]

The prediction of child behavior in a dental visit will help the dentist select a method to control behavior management. Many studies reported that birth order of children may influence their personality.[2] A review of birth order in the available published English literature recommends a relationship between different indices of cognitive ability, particularly verbal abilities.[3] Studies showed that birth-order had impacts on the intelligence[4] and personality.[5-6] Sulloway proposed a family dynamics model, concerning the birth order, claiming that it can influence the personality and behavior.[5-6] All model profiles, from the oldest to the youngest child, showed certain characteristics which were consistent with their birth order in the family system. The first-born children are the center of attention and care,[7] although susceptible to stress and uncertain in difficult circumstances.[8] They represent adult attitudes, such as seriousness, controlling, being organized and target-oriented.

The only child represents some of the personality traits of the firstborn, mainly the high achiever’s attitude, although they have their own particular personality traits. The only child can present defensiveness, desire innovation, perfectionism, self-confidence, controlling, rational, and intellectual behaviors.[1, 6] However, there are still controversies over this issue.[9-12] To the best of our knowledge, just one published study evaluated the effect of birth order on the children behavior in dental settings.[13]

This study was designed to evaluate how the children’s ordinal position influences their behavior in dental measurements. 

## Materials and Method

The present study was a randomized triple-blinded cross-sectional study. The subjects consisted of 158 healthy (ASA 1) 5-7 years old children referring to dental clinic in downtown Shiraz, Iran. All participants had at least one primary mandibular molar which needed class (I) restoration. The inclusion criteria were negative history of either any previous dental treatment or previous unpleasant medical experience.

The consent forms were granted by the parents after receiving a complete explanation of the study procedures. Baseline data, birth order, and number of children were asked from the parents. Then, the children were classified based on ordinal position as first-born, middle, youngest, and single child. This selection displayed in what ordinal position the participant was. The middle position was described as having siblings (sister or brother) before and after (irrespective of the number of children). It was described as if the child had at least one sister or brother after and one sibling before in the family. 

The anesthetic injection procedure was carried out by an academic pediatric dentist. A blind assessor, who was unaware of child's position in the family, asked the patients to rate their pain on visual analogue scale (VAS) diagram after injection. The assessor also objectively recorded the sound, eye, and movement (SEM) during the injections (Table 1). Total scores for SEM ranged from 0 to 9 based on 0–3 score for each parameter. For a robust assessment of the child's anxiety, two assessment methods; behavioral and self-report; were employed.

**Figure 1:**
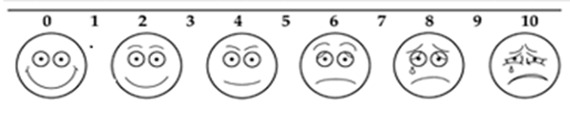
Visual Analogue Scale (VAS)

Using VAS assessment method (Figure 1), the subjects were instructed how to point their fingers to the position on the line between faces to indicate the amount of pain they felt. In this system, a 100-mm long row, ranging from very happy to very unhappy face was used. The total scores of perceiving pain ranged from 0 to 100. The children were invited to indicate their response on the row of faces. The pain perception was recorded based on measuring the distance (in millimeters) from the left end bar to the point that was mentioned by child. Higher scores indicated more severe pain.[14]

**Children's Behavior Questionnaire**

For assessment of the child anxiety, we employed Dental Subscale of the Children's Fear Survey Schedule (CFSS-DS) which is made to appraise fear of children in dental procedures.[15-16] This scale consists of 15 items to be responded with a 5-point scale which ranges from number 1 (not afraid at all) to number 5 (very afraid). This scaling is related to different characteristics of dental therapy such as invasive dental treatments (injections and drilling) and also to more general medical procedures. Total scores consequently would range from 15 to 75 and a score of 38 or more would be associated with clinical dental fear.[15]

During the trans-cultural adaptation of the CFSS-DS, only slight cultural adaptations were necessary. In any case, one examiner was in attendance when children were carrying out the measures. The items on the paper were read out loud and explained. The validity and reliability of the translated trial were confirmed.[17]

## Result

The present study revealed that 36 children (%23) were only child and 122 had siblings (%77), 30 were first-born (%19), 46 were middle (%29), and 46 were last-born (%29).

The results of this study showed that the mean difference of VAS, SEM scales, and anxiety were statistically significant among the children based on birth order (*p*< 0.001) (Table 1a and 1b).

**Table 1a:** Comparison of the mean SEM scale between the single child and those with siblings

**Birth Order**	**Frequency**	**Mean SEM Score**	**SD**
Single child	36	6.33	0.78
Children with siblings	122	3.51	1.76
Total	158	4.41	2.17

**Table 1b:** Comparison of the mean VAS between the single child and children with siblings

**Birth Order**	**Frequency**	**Mean VAS Score**	**SD**
Single	36	53.47	16.76
Children with siblings	122	38.45	20.65
Total	158	34.04	14.63

The results revealed that single children were significantly less cooperative and more anxious than the other children (*p*< 0.001) (Table 1c).

**Table 1c:** Comparison of the mean Stress between the single child and children with siblings

**Birth Order**	**Frequency**	**Mean Stress score**	**SD**
Single child	36	35	10.23
Children with siblings	122	26.49	9.01
Total	158	27.80	12.23

The middle children were significantly more cooperative in comparison with those in other position (*p*< 0.001) (Table 2a, 2b and 2c).

**Table 2a:** Comparison of the mean VAS scale between the middle child and those with other birth orders

**Birth Order**	**Frequency**	**SEM**	**SD**
Middle child	46	2.03	0.79
Children with other birth order	112	5.37	1.95
Total	158	4.41	2.17

**Table 2b:** Comparison of the mean VAS scale between the middle child and those with other birth orders

**Birth Order**	**Frequency**	**VAS**	**SD**
Middle Children	46	20.37	11.75
Children with other birth order	112	52.43	19.14
Total	158	34.04	14.63

**Table 2c:** Comparison of the mean Stress scale between the middle child and children with other birth orders

	**Frequency**	**Stress Scale**	**SD**
Middle children	46	19.85	5.08
Children with other birth order	112	33.75	9.21
Total	158	27.80	12.23

Data analysis found that the middle children showed significantly lower anxiety in dental setting than the other children (*p*< 0.001) (Table 2c).

The correlation test revealed a significant relation between anxiety and behavior based on SEM and VAS scales and Stress Score, respectively (t=0.93, *p*< 0.001; t=0.75 *p*> 0.001; and t=0.75, *p*< 0.001).

## Discussion

The study showed that single children exhibit more problem and anxiety in dental procedures compared to other children. Single-born children are at the core centre of attention and care. Even though they adopt adult characteristics, they are vulnerable to stressful situations and uncertain in difficult positions. Dental practice is recognized as one of these stressful situations that children may confront during their life.[13]

A study enrolled by Aminabadi *et al.*[13] experienced that only children are more prone to develop anxiety and negative behavior than children who are not alone in the family. Their results, in line with our findings, showed that absence of siblings and higher closeness with adults could hinder the intellectual development in the personality and social adaptation.[13] It is reported that only children receive too much attention and show early maturity. Since they do not have any siblings, they become self-centered, demanding, needy and moody when they are compared with children who had siblings.[18]

Tavares *et al.* showed that single children tend to be more introverted than children who were born after siblings.[19] The reasons for these findings would be that parents may have higher expectations from their single kids, and thus, these children are more sensitive about social failures. Moreover, later-born children should acquire social skills more rapidly to confer their personal needs in their relationships among siblings.[19] Cameron *et al.* reported that single children showed more unenthusiastic behaviors and they were less trusting.[20] In the current study, the middle children were more cooperative and showed significantly less anxiety than other children.

The findings of some published researches were in accordance with our results.[21-23]Middle-born children had more positive views toward friends and friendship than first- and last-born children.[24] Some studies reported that the middle-born children were less anxious and could communicate more conveniently than other children in different conditions.[21-23] They might be either really quiet or sociable and active. Middle children discover the individual worthiness through interpersonal evaluation.[23] However, Aminabadi *et al.* stated that the first-born children would demonstrate less negative behavior than later-born children in the dental procedures.[13]

Salmon reported that the first-born children behaved influentially and powerfully; a finding that was in line with what was observed in the current study.[24] This might have been due to the different origin of children’s family that could have an imperative effect on their personalities.[25]

Biologically oriented explanations emphasize the biological reserves depletion of the younger parents, particularly mothers. It is explained that when the successive children are added to the family, it causes less desirable intrauterine as well as postnatal influences on the development of later-born.[10] Another competing hypothesis labeled 'resource dilution' is that parental resources are restricted and that siblings are competitors for parents' time, energy, and economic resources; hence, later-born children are competing for a smaller portion of the family reserves.[26] Although the findings are controversial, the majority of studies concluded that there was no significant association between birth order and personality. In agreement with the doubts proposed by Ernst and Angst about the birth-order influences, several studies could not support Sulloway's findings that claimed the family dynamics frame of birth order would affect personality and behavior.[27-28]

After evaluating most of the published research on the topic, Ernst and Angst concluded that birth order did not appear to have a very strong influence in building the personality in a particular way.[8] Investigation of the family environment as a source of variances in sibling personality may suggest an elucidation for the little effect of birth order. The researcher concluded that personality outcomes were influenced by a multiplicity of interacting environmental factors including parental intervention, peer relationships and family sib-ship size; any single influence is dubious to clarify much divergence.[28] The efforts to associate the birth order to the personality values have usually yielded unreliable results.[27]

Many researchers evaluated the effects of birth order on a family system. There was not much research out there on birth order and how it affects a person’s life, their relationships specifically. It seems that children behave differently in various situations and it may not be enclosed into a predictable pattern. The family atmosphere changes with the birth of each child. The children’s personality improves with the relationships they establish with their parents and their siblings. Children pass through many different life experiences and situations that would inevitably affect their personality. Although these contradictory findings can be explained by differences in the study designs, methods of data collection, and specific factors evaluated, biological factors may also play an important role in child nature. It was shown that the effects of biological birth-order, resulting mainly from intrauterine influences on personality, may in fact explain the inconsistencies of personality between siblings. Whether as mostly psychological, biological, or a mixture, birth order appears to be diffidently linked to the personality development.[29]

Birth-order influences should be regarded in combination with other factors such as gender, age dissimilarities between siblings, socioeconomic families, family environment, family morals, and culture.[30]

## Conclusion

The birth order of children may affect their behavior in dental measurements just as it influences their personality. Single children exhibit more problem and anxiety in dental procedures compared with other children. 

## References

[1] Frankl S, Shiere F, Fogels H (1962). Should the parent remain with the child in the dental operatory?. J Dent Child.

[2] Luecken LJ, Lemery KS (2004). Early caregiving and physiological stress responses. Clin Psychol Rev.

[3] Schultz D, Izard CE, Ackerman BP, Youngstrom EA (2001). Emotion knowledge in economically disadvantaged children: self-regulatory antecedents andrelations to social difficulties and withdrawal. Dev Psychopathol.

[4] Zajonc RB (2001). The family dynamics of intellectual development. Am Psychol.

[5] Paulhus DP, Trapnell D (1999). Chen Birth-order effects on personality and achievement within families. Psychological Science.

[6] Sulloway F, Holcomb HR (2001). Birth-order, sibling competition, and human behavior. Conceptual challenges in evolutionary psychology: Innovative research strategies.

[7] Richardson RW, Richardson LA (1990). Birth Order and You.

[8] Ernst C, Angst J (1983). Birth Order: Its influence on personality.

[9] Price J (2008). Parent-child quality time: Does birth order matter?. J Human Resources.

[10] Draper PH (2000). Birth order, and fertility among Ju/Hoansi (!Kung). Human Nature 2000, and 11:117-56., Birth order, sibling investment, and fertility among Ju/Hoansi (!Kung). Human Nature.

[11] Downey DB (2001). Number of siblings and intellectual development. The resource dilution explanation. Am Psychol.

[12] Skinner NF (2003). Birth order effects in dominance: failure to support Sulloway's view. Psychol Rep.

[13] Aminabadi NA, Sohrabi A, Erfanparast LK, Oskouei SG, Ajami BA (2011). Can birth order affect temperament, anxiety and behavior in 5 to 7-year-old children in the dentalsetting?. J Contemp Dent Pract.

[14] Buchanan H, Niven N (2002). Validation of a Facial Image Scale to assess child dental anxiety. Int J Paediatr Dent.

[15] Venham LL, Gaulin-Kremer E, Munster E, Bengston-Audia D, Cohan J (1980). Interval rating scales for children's dental anxiety and uncooperative behavior. Pediatr Dent.

[16] Rothbart MK, Ahadi SA, Hershey KL, Fisher P (2001). Investigations of temperament at three to seven years: the Children's Behavior Questionnaire. Child Dev.

[17] Javadinejad S, Farajzadegan Z, Madahain M (2011). Iranian version of a face version of the Modified Child Dental Anxiety Scale:Transcultural adaptation and reliability analysis. J Res Med Sci.

[18] Howarth E (1980). Birth order, family structure and personality variables. J Pers Assess.

[19] Tavares MB, Fuchs FC, Diligenti F, de Abreu JR, Rohde LA, Fuchs SC (2004). Behavioral characteristics of the only child vs first-born and children with siblings. Rev Bras Psiquiatr.

[20] Cameron L, Erkal N, Gangadharan L, Meng X (2013). Little emperors: behavioral impacts of China's One-Child Policy. Science.

[21] Schneider LJ, Reuterfors DL (1981). The impact of birth order and sex on social interest. J Individual Psychology.

[22] Schachter SL, Festinger DH (1995). The psychology of affiliation: Experimental studies of the sources of gregariousness.

[23] Kalkan M (2008). The Relationship of Psychological Birth Order to Irrational Relationship Beliefs. Social Behaviour and Personality.

[24] Salmon C (2003). Birth order and relationships: Family, friends, and sexual partners. Hum Nat.

[25] Guastello DD, Guastello SJ (2002). Birth category effects on the Gordon Personal Profile variables. JASNH.

[26] Downey DB (2001). Number of siblings and intellectual development. The resource dilution explanation. Am Psychol.

[27] Jefferson T, Herbst JH, McCrae RR (1998). Associations between birth order and personality traits: Evidence from self-reports and observer ratings. J Res Personality.

[28] Michalski RL, Shackelford TK (2002). An attempted replication of the relationships between birth order and personality. Journal for Research on Personality.

[29] McGowan H, Beck EA (2009). Qualitative investigation of middle siblings. TCNJ J Student Scholarship.

[30] Eckstein D (2000). Empirical studies indicating significant birth-order related personality differences (Electronic version). Journal of Individual Psychology.

